# CIRCpedia v2: An Updated Database for Comprehensive Circular RNA Annotation and Expression Comparison

**DOI:** 10.1016/j.gpb.2018.08.001

**Published:** 2018-08-29

**Authors:** Rui Dong, Xu-Kai Ma, Guo-Wei Li, Li Yang

**Affiliations:** 1 CAS Key Laboratory of Computational Biology, CAS-MPG Partner Institute for Computational Biology, Shanghai Institute of Nutrition and Health, Shanghai Institutes for Biological Sciences, University of Chinese Academy of Sciences, Chinese Academy of Sciences, Shanghai 200031, China; 2 School of Life Science and Technology, ShanghaiTech University, Shanghai 201210, China

**Keywords:** Circular RNA, CircRNA, Back-splicing, Database, CIRCpedia

## Abstract

**Circular RNAs** (**circRNAs**) from **back-splicing** of exon(s) have been recently identified to be broadly expressed in eukaryotes, in tissue- and species-specific manners. Although functions of most circRNAs remain elusive, some circRNAs are shown to be functional in gene expression regulation and potentially relate to diseases. Due to their stability, circRNAs can also be used as biomarkers for diagnosis. Profiling circRNAs by integrating their expression among different samples thus provides molecular basis for further functional study of circRNAs and their potential application in clinic. Here, we report **CIRCpedia** v2, an updated **database** for comprehensive circRNA annotation from over 180 RNA-seq datasets across six different species. This atlas allows users to search, browse, and download circRNAs with expression features in various cell types/tissues, including disease samples. In addition, the updated database incorporates conservation analysis of circRNAs between humans and mice. Finally, the web interface also contains computational tools to compare circRNA expression among samples. CIRCpedia v2 is accessible at http://www.picb.ac.cn/rnomics/circpedia.

## Introduction

Eukaryotic mRNA precursors (pre-mRNAs) usually undergo splicing to remove introns and join exons by ligating an upstream 5′ splice site (5′ ss) with a downstream 3′ ss to produce linear RNAs with 5′ to 3′ polarity. Splicing can also happen in a reversed order to ligate a downstream 5′ ss with an upstream 3′ ss, referred to as back-splicing. Back-splicing of exon(s) leads to the formation of circular RNAs (circRNAs), featured by covalently close circle structure in nature [1], [2], [3], [4], [5]. Although discovered more than 25 years ago in higher eukaryotes, only a few circRNAs were found at that time, which were thought as byproducts from mis-splicing with little functional potential [6], [7], [8], [9], [10]. However, recent genome-wide profiling of nonpolyadenylated RNA transcriptomes with specific computational approaches that aim to identify reads mapped to back-splicing junctions, has identified a large number of circRNAs in various cell lines/tissues and across different species [11], [12], [13], [14], [15], [16], [17], [18], [19].

Recent research progresses have shown that back-splicing is catalyzed by canonical spliceosomal machinery [20], [21], [22], but in a relatively low efficiency [23], possibly due to the unfavorable spliceosome assembly at back-splicing junction sites. The formation of circRNAs can be regulated by both *cis*-elements and *trans*-factors. On the one hand, orientation-opposite complementary sequences, when located pairwise in introns flanking circRNA-producing exons, can facilitate back-splicing for circRNA formation. On the other hand, RNA binding proteins can associate with intronic *cis*-elements/sequences to further regulate circRNA biogenesis [16], [24], [25], [26], [27]. Recent studies have shown that some circRNAs get involved in gene expression networks through a variety of molecular mechanisms, such as competing with canonical splicing, sequestering microRNAs/proteins or interfering with RNA polymerase II-mediated transcription [13], [24], [27], [28], [29], [30]. These new findings thus suggest that circRNAs, at least some of them, can act as regulatory RNA molecules. Therefore, comprehensive annotation of circRNAs will likely provide molecular basis for understanding the function of circRNAs and their potential applications in clinic.

Although generally expressed at low levels, circRNAs are highly enriched in brains/neurons [18], [23], [31]. This phenomenon can be partially explained by the resistance of circRNAs to exonucleolytic degradation, which allows these RNA circles to gradually accumulate to relatively high levels in cells with slow division rates, such as neurons [23]. Interestingly, significantly more circRNAs are detected in humans than in mice, fruitflies, and worms [32]. As produced from annotated protein-coding genes, sequence conservation of circRNA-producing exons is similar to that of their neighboring linear RNA-producing exons across humans and mice [18], [33]. However, only a small portion (10%–20%) of human circRNAs could be observed in mouse samples [18], [32], [33], which is likely due to the different composition of orientation-opposite complementary sequences across species [32]. It has been shown that short interspersed nuclear elements (*SINE*s) and primate-specific *Alu*s, when juxtaposed in introns flanking circRNA-forming exons, contribute the most to the circRNA formation. The complementarity of *Alu* elements in humans is much higher than those of *SINE*s or other complementary sequences in mice, fruitflies, and worms, which may lead to the observed species-specific circRNA expression [18], [32].

Despite the diversified circRNA expression across cell lines/tissues and species, multiple circRNAs can be produced by different selections of alternative back-splicing and/or canonical alternative splicing within circRNAs [34], [35]. Competition of putative RNA pairs formed by orientation-opposite complementary sequences across different sets of introns that bracket alternative back-splice sites can lead to alternative back-splicing selection [35], which is largely diverse among the cell lines/tissues examined. In this case, identification of different alternative back-splicing events serves as a basis to study their cell type-specific and tissue-specific functions.

Here, we report CIRCpedia v2, an updated database for comprehensive circRNA annotation from over 180 RNA-seq datasets across six different species. This atlas allows users to search, browse, and download circRNAs and alternative back-splicing events with expression characteristics/features in various cell types/tissues, including disease samples. The updated database also provides conservation information of circRNAs between humans and mice after LiftOver analysis. Finally, the web interface also contains computational tools for simple comparison of circRNA expression between samples. CIRCpedia v2 is accessible at http://www.picb.ac.cn/rnomics/circpedia.

## Implementation

CIRCpedia v2 is implemented with Flask (a lightweight framework designed to build web applications; http://flask.pocoo.org/), Bootstrap (an open source front-end framework for developing web pages; https://getbootstrap.com/), jQuery (a library designed to simplify the client-side javascript programming; https://jquery.com/), Bokeh (a python library for interactive data visualization; https://bokeh.pydata.org/en/latest/), and JBrowse (a genome browser for visualizing track files; https://jbrowse.org/). SQLite (a structured query language database engine; https://www.sqlite.org/index.html) is used to store metadata information. Codes are developed by using Visual Studio Code (a flexible and customizable source code editor that supports a series of programming languages; https://code.visualstudio.com/). Finally, CIRCpedia v2 is hosted on a Red Hat Enterprise Linux system with Apache HTTP Server to provide a stable web access.

## Database content and usage

### Summary of new features

**Figure 1:**
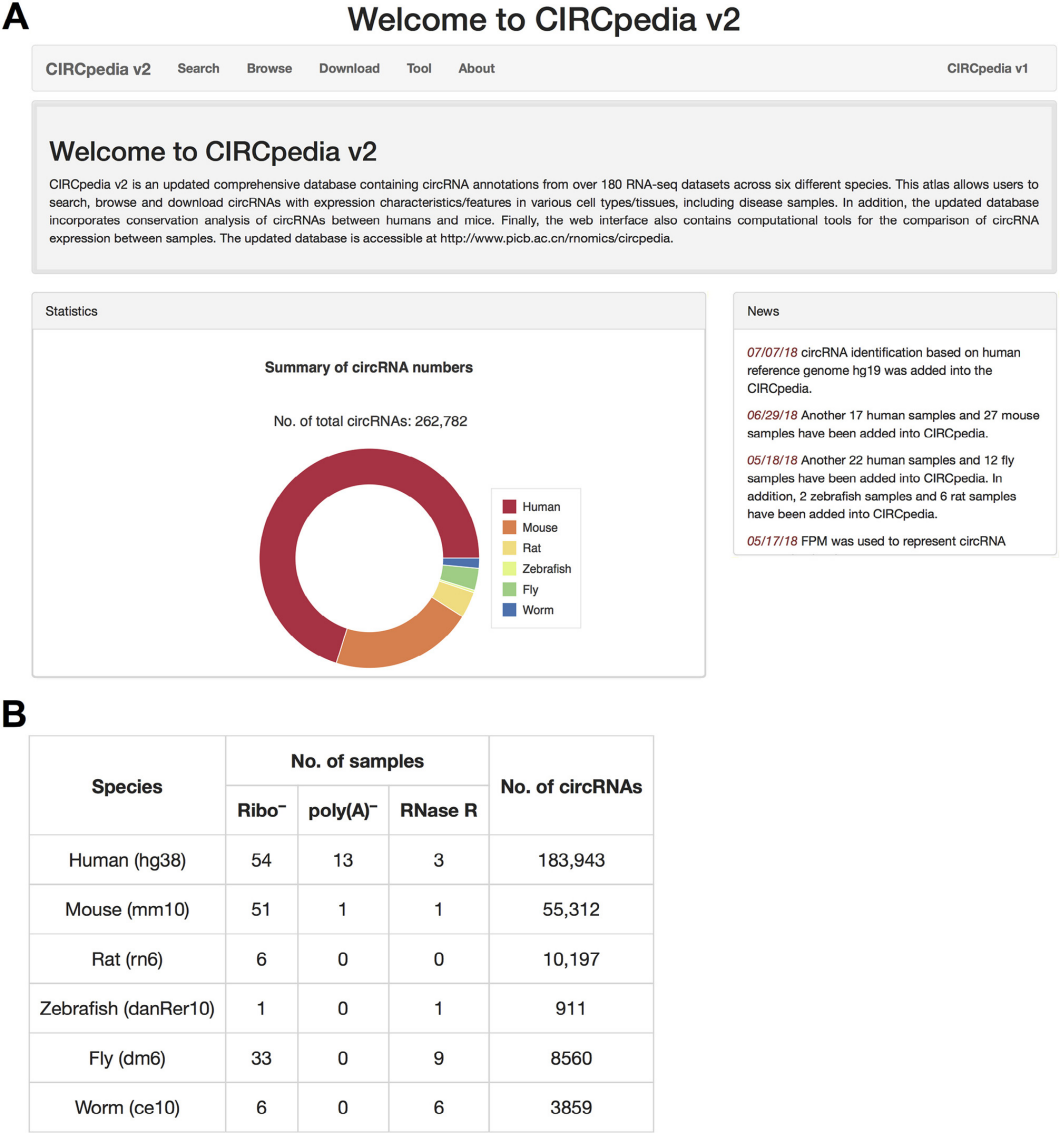
**Homepage of CIRCpedia v2** **A.** Summary of annotated circRNAs in CIRCpedia v2. **B.** Datasets used in CIRCpedia v2.

CIRCpedia v2 significantly expands the data contents by collecting various RNA-seq datasets from the Gene Expression Omnibus (GEO), Encyclopedia of DNA Elements (ENCODE) project, and the EMBL-European Bioinformatics Institute (EMBL-EBI) database. Compared to the previous version [35], CIRCpedia v2 includes a comprehensive annotation of circRNAs and alternative back-splicing events by analyzing additional cell lines/tissues and species (Figure 1A). We have applied fragments mapped to back-spliced junctions per million mapped fragments (FPM) value to quantitate circRNA expression, which is suitable for both single-read and paired-end sequencing datasets. CIRCpedia v2 incorporates conservation information of circRNAs between humans and mice by LiftOver analysis [32]. In addition, a new version of JBrowse is embedded for circRNA visualization. Finally, a new web interface for direct circRNA expression comparison among different samples is incorporated into CIRCpedia v2, labeled as “Tool”. In summary, CIRCpedia v2 is a comprehensive, user-friendly, and powerful database with circRNA search, visualization, downloading, and online analysis.

### Data statistics of CIRCpedia v2

Current version of CIRCpedia v2 contains 185 datasets (86 new ones compared with those in CIRCpedia) from ribo^−^, poly(A)^−^, ribo^−^/RNase R, or poly(A)^−^/RNase R RNA-seq (Figure 1B). Among these 86 new datasets, 39 samples are from humans, 27 are from mice, and 12 are from fruitflies, respectively. In addition to humans, mice, fruitflies, and worms that have been analyzed in early version, datasets from two additional species are also incorporated in CIRCpedia v2, including six ribo^−^ RNA-seq datasets from rats, as well as one ribo^−^ RNA-seq and one ribo^−^/RNase R RNA-seq datasets from zebrafish. After identifying RNA-seq reads that are mapped to back-splicing junction sites by CIRCexplorer2 [35] (http://circexplorer2.readthedocs.io/en/latest/), a circRNA annotation track file (BED), an alternative 5′ back-splicing (A5BS) annotation track file (BED) and an alternative 3′ back-splicing (A3BS) annotation track file (BED) are obtained for each RNA-seq dataset. In total, about 262,782 back-splicing events, which represent at least 262,782 circRNAs, are annotated in CIRCpedia v2 in the samples examined (Figure 1). About 39,225 A5BS and 34,747 A3BS events are also included in CIRCpedia v2. Expression levels of all back-splicing events (including A5BS and A3BS) are indicated using FPMs.

### Web interface for search

**Figure 2:**
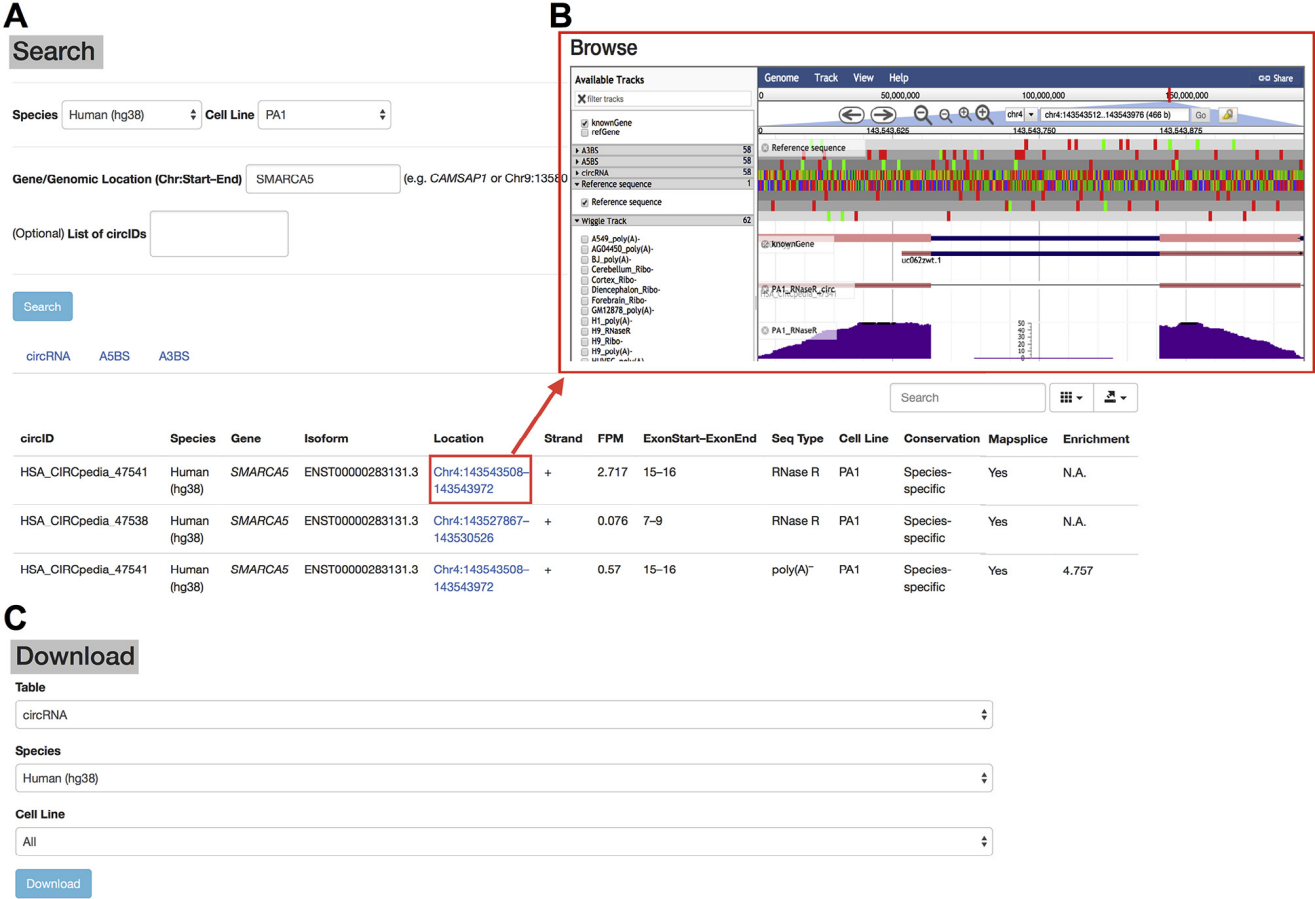
**Screenshots of web interface in CIRCpedia v2** **A.** Search circRNAs with query. The result table shows the annotation of circRNAs originating from human *SMARCA5* locus. **B.** Browse circRNAs by JBrowse visualization. **C.** Download circRNAs for further analysis.

CIRCpedia v2 provides a “Search” function by querying genomic locations, gene symbols, or CIRCpedia IDs (circIDs) (Figure 2A). Search using a specific gene symbol (genomic location) can retrieve all circRNA and related alternative back-splicing events in this locus (/genomic location). Output table files include circID, species, gene, isoform, location, strand, FPM, ExonStart-ExonEnd, seq type, cell line, conservation between humans and mice, information on annotation by MapSplice alignment, and enrichment fold change after RNase R treatment. Here, the conservation information is obtained by using LiftOver tool to identify conserved circRNAs between humans and mice (Methods). Notably, other than CIRCexplorer2 [16], [35], another computational pipeline, MapSplice [36], is also used for human circRNA annotation. In addition, when applicable, enrichment fold change is also listed in ribo^−^ and poly(A)^−^ samples with corresponding RNase R treatment. Meanwhile, output files for A5BS and A3BS by the same queries are available, listed with the information of species, gene, isoform, location, strand, percent circularized-site usage (PCU), sequencing type, and cell line. Users can limit their queries to specific species or cell line/tissue using different setting options (Figure 2A). The output table files can be exported in different formats, such as JSON, XML, CSV, or TXT. Useful links are also available for circRNA visualization (see below) and host gene identification at GeneCards websites (for humans only).

### Web interfaces for browse and downloading

A “Browse” function is also available in CIRCpedia v2. After “Search”, the identified circRNAs can be directly linked to the website-embedded JBrowse for visualization (Figure 2B). Alternatively, users can visualize circRNAs by selecting different options on the page of “Browse”. Different types of tracks, including gene annotation, RNA-seq dataset, circRNA annotation, and alternative back-splicing event, are available in the left panel of JBrowse, with the selected tracks shown in the right panel. Users can jump between different species by clicking “Genome” button at the top left corner of JBrowse. Furthermore, by clicking the track of a given circRNA in JBrowse, its expression value (FPM) pops up for visualization.

With the “Download” function, tables of circRNAs and alternative back-splicing events from the selected cell lines/tissues and species can be retrieved from CIRCpedia v2 (Figure 2C). The output files are in CSV format, listed with the information of circID, species, gene, isoform, location, strand, FPM, ExonStart-ExonEnd, sequencing type, cell line, conservation, information on annotation by MapSplice alignment, and enrichment fold change after RNase R treatment.

### Web interface for circRNA expression analysis

**Figure 3:**
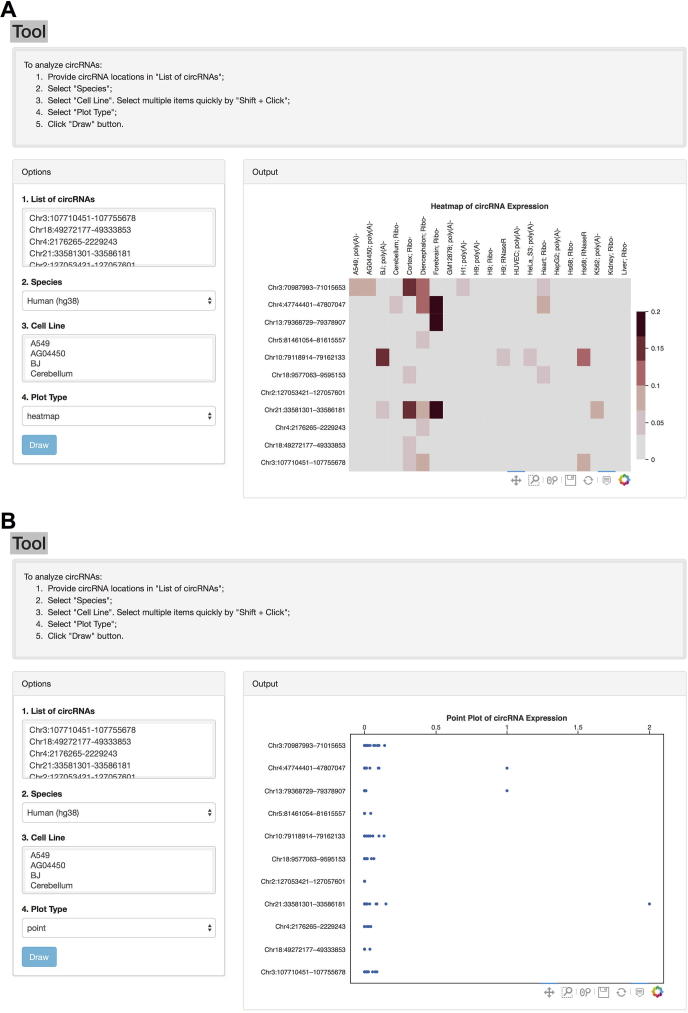
**Screenshots of a new “Tool” interface for direct online analysis** **A.** Heatmap to show differential circRNA expression among selected samples. **B.** Point plot to show differential circRNA expression among selected samples.

A new “Tool” function is now available in the web interface of CIRCpedia v2. A list of circRNAs with their genomic locations can be directly copied and pasted for expression comparison analysis. Currently, CIRCpedia v2 enables two types of data visualization, heatmap and point plot (Figure 3). As shown in Figure 3A, a heatmap diagram is generated to show differential circRNA expression (FPM values) in selected cell lines. Alternatively, users can choose a different method, such as point plot, to illustrate differential circRNA expression among various samples (Figure 3B).

## Discussion and perspectives

Without 3′ polyadenylated tails, circRNAs had been largely missed by the enrichment of polyadenylated RNAs for deep sequencing (referred to as poly(A)^+^ RNA-seq), but were successfully captured by two nonpolyadenylated RNA isolation strategies prior to deep sequencing (referred to as ribo^−^ or poly(A)^−^ RNA-seq, respectively) [16], [35]. The ribo^−^ RNA-seq profiles both nonpolyadenylated and polyadenylated transcripts after depleting rRNAs, and the poly(A)^−^ RNA-seq examines relatively pure nonpolyadenylated transcripts by getting rid of both rRNAs and polyadenylated RNAs. With the advent of deep sequencing for nonpolyadenylated RNAs, a large number of circRNAs from back-splicing of exons have been widely identified in various cell lines/tissues and across distinct species. A comprehensive annotation of circRNAs is necessary for further circRNA research.

Here, we update CIRCpedia database to version 2 by providing comprehensive circRNA annotation and online tool for circRNA expression analysis. We integrate “Search”, “Browse”, “Download”, and “Tool” functions into the web interface. Users can search circRNAs by querying specific genomic regions or gene names. Meanwhile, tracks of circRNAs, alternative back-splicing events, and RNA-seq files can be visualized by embedded JBrowse directly in the web interface. With the “Download” function, information of circRNAs and alternative back-splicing events from selected cell lines/tissues and species can be retrieved for additional analysis. Finally, users with limited bioinformatics background can choose a new online “Tool” function for direct comparison of circRNA expression among different cell lines/tissues. Compared to other circRNA databases, such as circBase [37], CSCD [38], or circRNAdb [39], CIRCpedia v2 provides a more comprehensive annotation of circRNAs with some unique characteristics. For instance, it contains 185 RNA-seq datasets, covering a wide range of cell lines/tissues and species, and leads to the identification of over 260,000 circRNAs from the transcriptomes examined. In addition, conservation analysis of circRNAs expressed across humans and mice is also included in CIRCpedia v2. Finally, CIRCpedia v2 provides useful bioinformatic tools for direct online comparison of circRNA expression.

We will continue to improve and update this circRNA atlas for the research community. When additional RNA-seq datasets are available, they will be incorporated into this database. Given combination of different circRNA prediction algorithms in transcriptome-wide profiling can reduce false positives [40], we also use other pipelines, such as MapSplice [36], to provide additional information for circRNA prediction. Currently, all datasets have been screened using CIRCexplorer2, and all human RNA-seq datasets have also been analyzed using MapSplice. circRNAs predicted using at least two methods will be further highlighted in CIRCexplorer2. Other useful online tools, such as calculating pairing capacity of orientation-opposite RNA complementary sequences [32], will also be introduced to CIRCpedia v2.

Taken together, we report here CIRCpedia v2, an updated circRNA database with integrated information of circRNA annotation, expression, and conservation. The CIRCpedia v2 database is freely available at http://www.picb.ac.cn/rnomics/circpedia.

## Methods

### Database design

CIRCpedia v2 contains circRNA tables, gene annotation tables, alternative back-splicing tables, and conservation tables. All the tables were stored in SQLite database. The pages for “Search” and “Download” are more convenient for cell line selection in specific species. JBrowse (version: 1.14.0) is embedded into CIRCpedia v2. In addition, a useful tool to compare circRNA expression is incorporated in the “Tool” page. Expression levels of circRNAs can be drawn by heatmap and point plot.

### Annotation and alternative back-splicing analysis of circRNAs

In total, 185 RNA-seq datasets were retrieved from GEO (https://www.ncbi.nlm.nih.gov/geo/), ENCODE (https://www.encodeproject.org/) project, and EMBL-EBI (https://www.ebi.ac.uk/) database across six species for analysis in this study. CIRCexplorer2 [16], [35] was used to retrieve RNA-seq reads that are mapped to back-splicing junction sites for circRNA prediction. Briefly, RNA-seq reads were mapped to reference genomes (human: GRCh38/hg38 and GRCh37/hg19; mouse: GRCm38/mm10; rat: RGSC 6.0/rn6; zebrafish: GRCz10/danRer10; fly: BDGP Release 6 + ISO1 MT/dm6; and worm: WS220/ce10) using HISAT2 (2.1.0, parameter: --no-softclip --score-min L, -16,0 --mp 7,7 --rfg 0,7 --rdg 0,7 --dta-cufflinks -k 1 --max-seeds 20). The unmapped reads were then further mapped to relevant genomes using TopHat-Fusion (version: tophat-2.0.12, parameter: --fusion-search --keep-fasta-order --bowtie1 --no-coverage-search). Reads mapped to back-splicing junction sites identified using TopHat-Fusion were further retrieved using CIRCexplorer2 pipeline based on known gene annotations (human (hg38): gencode.v27.annotation.gtf and refFlat.txt updated at 2017/08/23; human (hg19): refFlat.txt updated at 2016/01/04, knownGene.txt updated at 2013/01/30 and ensGene.txt updated at 2014/04/06; mouse (mm10): refFlat.txt 2016/06/04, knownGene.txt 2015/06/01 and ensGene.txt updated at 2014/04/06; rat: refFlat.txt updated at 2018/01/01 and ensGene.txt updated at 2017/09/03; zebrafish (danRer10): refFlat.txt updated at 2018/02/25 and ensGene.txt 2018/02/04; fly (dm6): refFlat.txt updated at 2017/05/28 and ensGene.txt updated at 2018/02/04; worm (ce10): refFlat.txt updated at 2013/03/18 and ensGene.txt updated at 2012/06/05) for circRNA annotation. Alternative back-splicing was analyzed using CIRCexplorer2 with default parameters [35]. For each of the RNA-seq datasets analyzed, a circRNA annotation track file (BED), an A5BS annotation track file (BED), and an A3BS annotation track file (BED) were generated after CIRCexplorer2 analysis. Another circRNA prediction pipeline, MapSplice (version: 2.1.8, parameter: --fusion --min-fusion-distance 200) [36], was also used for human circRNA annotation. The expression level of circRNAs was evaluated with FPM.

### Conservation analysis

In order to obtain the circRNAs conserved between humans and mice, LiftOver tool (parameters: -bedPlus=3 -tab -minMatch=0.1 -minBlocks=1) was used to identify orthologous back-splicing sites between humans and mice. For each human circRNA, the circRNA is defined as a conserved circRNA between humans and mice, if it is expressed in mouse orthologous locus within 5-nt difference; otherwise, the circRNA would be considered as human-specific, if no mouse circRNA ortholog is found. Similar pipeline was also applied to mouse circRNAs. It is worth noting that more conserved circRNAs are expected to be identified if additional datasets are available for this analysis in the future.

## Authors’ contributions

LY conceived and designed the project. RD, XKM, and GWL collected the data. RD analyzed the data with the help from XKM and GWL. XKM designed the website with the help from RD. LY wrote the paper with inputs from all authors. All authors read and approved the final manuscript.

## Competing interests

The authors declare no competing financial interests.
